# Carboxymethyl Chitosan Hydrogels for Effective Wound Healing—An Animal Study

**DOI:** 10.3390/jfb14090473

**Published:** 2023-09-15

**Authors:** Karol Kamil Kłosiński, Radosław Aleksander Wach, Weronika Kruczkowska, Łukasz Duda, Damian Kołat, Żaneta Kałuzińska-Kołat, Piotr Tomasz Arkuszewski, Zbigniew Włodzimierz Pasieka

**Affiliations:** 1 Department of Biomedicine and Experimental Surgery, Faculty of Medicine, Medical University of Lodz, Narutowicza 60, 90-136 Lodz, Poland; lukasz.duda@umed.lodz.pl (Ł.D.); damian.kolat@umed.lodz.pl (D.K.); zaneta.kaluzinska@umed.lodz.pl (Ż.K.-K.); piotr.tomasz.arkuszewski@umed.lodz.pl (P.T.A.); zbigniew.pasieka@umed.lodz.pl (Z.W.P.); 2 Institute of Applied Radiation Chemistry, Faculty of Chemistry, Lodz University of Technology, Wróblewskiego 15, 93-590 Lodz, Poland; 3 Faculty of Biomedical Sciences, Medical University of Lodz, Żeligowskiego 7/9, 90-752 Lodz, Poland; weronika.kruczkowska@stud.umed.lodz.pl; 4 Department of Functional Genomics, Faculty of Medicine, Medical University of Lodz, Żeligowskiego 7/9, 90-752 Lodz, Poland

**Keywords:** polysaccharide hydrogel, PEGDA, radiation crosslinking, chronic wound, wound histopathology, rat model

## Abstract

Hydrogels have various applications in medicine, for example, in systems for controlled drug release or as wound dressings, where they provide an appropriate environment for healing and constitute a barrier to microorganisms. The aim of this study was to evaluate the action of carboxymethyl chitosan (CMCS) hydrogels in wound healing therapy in vivo using a laboratory rat model. The hydrogels were formed from aqueous solutions of a CMCS biopolymer via electron beam irradiation, with the presence of a crosslinking agent of poly(ethylene glycol) diacrylate. A histopathological examination of injured tissue, using a model of a hard-to-heal wound, indicated that the CMCS hydrogel supported healing. The new gel dressing, being noncytotoxic, presents great potential in wound treatment, with positive effects on the amount of inflammatory infiltration, young collagen formation, and the degree of epidermalization. A key advantage of the current approach (i.e., using competitive radiation technology for synthesis) is that it includes only one step, with the product being sterilized as it is synthesized. The hydrogel effectively supports wound healing and can serve as a bio-based and biodegradable platform for other medical applications.

## 1. Introduction

The mechanisms that occur in the human body at a wound site are relatively well understood and involve various inflammatory mediators, as well as several specific cell types. If the wound healing process proceeds properly, the tissue returns to its pre-injury state. However, in some cases, the process may result in chronic wounds that can be accompanied by infections [1,2]. It is assumed that in developed countries 1–2% of the population will experience a chronic wound in their lifetime, and these numbers are gradually increasing. This is related to aging populations, as proper wound closure is negatively correlated with patient age [3]. Chronic wounds have several critical complications, including hemorrhage, sepsis, and amputation [1,3]. Various approaches to chronic wound management are currently in use, but none of them seem to be thoroughly satisfying.

Hydrogels have a number of advantages that support wound healing. Different types may be distinguished based on their composition, source of components, preparation method, character (permanent chemical hydrogels or those stabilized using physical interactions), physicochemical properties (e.g., ionic charge, strength, and stimuli-responsive character), and biological responsiveness [4]. In medical applications, hydrogels have been studied for use as dressings in, inter alia, acute burn treatment, diabetic foot ulcer management, and oral mucositis [5,6,7,8,9]. In vivo studies have shown that rats demonstrate faster tissue regeneration upon the application of hydrogel dressings, as well as increased collagen deposition and no scar formation [7]. Despite the large number of hydrogel dressings available on the market, there is still the need to develop materials that accelerate wound healing. The unique therapeutic effect of hydrogels, in addition to providing a proper environment, may be enhanced by their composition; the use of natural polymers as the main components of hydrogels improves their biological activity, biocompatibility, and biodegradability [4,10]. An interesting example of a hydrogel dressing containing natural polymers is the multicomponent material PEGS-PBA-BA/CS-DA-LAG (phenylboronic acid and benzaldehyde bifunctional polyethylene glycol-co-poly(glycerol sebacic acid), dihydrocaffeic acid, and L-arginine cografted chitosan), which promotes the healing of type II diabetic foot; treatment was found to reduce inflammation and increase angiogenesis based on the re-epithelization rate, wound closure rate, or regeneration of blood vessels and vesicles, as well as collagen metabolism [11].

One natural polysaccharide with a broad history in the medical field is chitosan (CS), a deacetylated product of chitin that is mostly obtained from crustacean shells. CS is composed mainly of D-glucosamine units, with some fraction of N-acetyl-D-glucosamine, connected by β-(1-4) bonds [12,13]. CS is reported to have excellent wound healing [4], antibacterial, and anti-inflammatory properties, as well as to demonstrate good biocompatibility and biodegradability. In addition, the polymer bears a positive charge that triggers erythrocyte adhesion, fibrinogen adsorption, and platelet activation, thus improving its hemostatic potential [14,15]. It is not only the subject of multidisciplinary research, but it is also in use in commercial medical devices dedicated for wound healing. One disadvantage of CS is its poor solubility at physiological pH (pH > 6), which may complicate its processing, limiting the use of this polysaccharide. In order to overcome this, various derivatives of this compound are being studied, such as chitosan esters or chitosan ethers [10,16,17]. One such example is carboxymethyl chitosan (CMCS), comprising carboxyl (-COOH) groups, next to native hydroxyl (-OH) and amine (-NH_2_) moieties. CMCS is also characterized by high biocompatibility and antimicrobial properties, but it has a broader solubility range in water than chitosan. Because of its advantages, CMCS may find use in drug delivery systems, biomedicine, or adsorption [18,19,20], as well as hydrogel material.

CMCS hydrogels can be obtained with classical chemical methods using crosslinkers or with radiation-induced crosslinking [21,22]. In our previous research, CMCS hydrogels were formed using radiation either with or without an efficient crosslinking agent, i.e., poly(ethylene glycol) diacrylate (PEGDA). It was demonstrated that ionizing radiation is a suitable tool for the synthesis of carboxymethyl chitosan-based hydrogels [23]. In addition, a new generation of carboxymethyl chitosan hydrogels formed with ionizing radiation have demonstrated their biocompatibility in vitro and in vivo [10,24].

Despite numerous studies on hydrogels, we assumed that their potential has not been fully exhausted. In addition to incorporating active compounds within the network [25], it is possible to control the healing process through the inherent properties of the network itself, i.e., using a chitosan-based network. Therefore, the main purpose of the current study was to evaluate in vivo the suitability and effectiveness of CMCS hydrogels in wound healing. This article aimed to answer the question of whether the hydrogel of this biopolymer can essentially be used instead of commercial hydrogel dressing using synthetic polymer. The added value may be its biological character resembling that of chitosan, its biodegradability, and its controlled absorption properties as an ampholyte. This study used a new CMCS hydrogel formed with radiation, which simultaneously synthesized and sterilized the polymer network, and demonstrates its wound healing potential under operational conditions.

## 2. Materials and Methods

### 2.1. Chemicals

Carboxymethyl chitosan (CMCS) was purchased from Kraeber & Co GmbH, Ellerbek, Germany. The viscosity- and weight-averaged molecular weights, respectively, were 350 kg∙mol^−1^ and 440 kg∙mol^−1^ (the latter was determined with light scattering); its deacetylation degree was 93.8%, and the substitution degree was 96%. High-purity poly(ethylene glycol) diacrylate (PEGDA; molar mass of 700 g·mol^−1^) and sodium perchlorate monohydrate (NaClO_4_·H_2_O) were purchased from Merck Life Sciences Sigma-Aldrich, Darmstadt, Germany. High-purity water (0.055 µS/cm; TKA MicroPure system, Thermo, Niederebert, Germany) was used in all of the experiments.

### 2.2. Hydrogel Manufacturing

Three solutions of 5% CMCS were prepared by dissolving the appropriate amount of polymer in a 0.075 mol·dm^−3^ solution of NaClO_4_ and stirring at room temperature for 72 h [24]. Perchlorate, a radiation-inactive salt, was used to reduce the mutually repulsive ionic or electrostatic interactions in the CMCS-ionized groups that inhibit gel formation, i.e., macroradical recombination. After the polymer was completely dissolved, PEGDA was admixed to a concentration of 5% in the final polymer–water mixture. Approximately 1 g of polymer solution was placed in radiation-resistant plastic bags and heat-sealed. The samples were irradiated at room temperature with a 6 MeV electron beam (EB) from a linear accelerator (Elektronika ELU-6e, Elektronika, Moscow, Russia) to a total dose of 25 kGy. The dose rate was ca. 5 kGy·min^−1^, as determined with alanine dosimetry.

### 2.3. Animals

The in vivo studies were conducted on Wistar laboratory rats (male; body weight: 300–350 g; age: 10–12 weeks). The selection of rats as the animal model (the primary experimental species), the number of groups, the experimental design, and the assessment of the local response to the implanted materials was carried out in accordance with international PN-EN:ISO 10993-6:2016 standard [26]. The animals were kept in an experimental room in transparent cages made of polycarbonate, five animals per cage, and in controlled environmental conditions with an adjustable light cycle of 12/12 h, temperature of 20–24 °C, relative humidity of 45–65%, and 15–20 air changes per hour. The animals received standard complete livestock feed and drinking water ad libitum. The animals underwent procedures after a seven-day adaptation period.

### 2.4. Animal Experiments—Wound Healing

The aim of the experiment was to determine the effectiveness of carboxymethyl chitosan hydrogel dressings at accelerating wound healing using a chronic wound model and to demonstrate their effectiveness at supporting wound healing. Laboratory rats were used as subjects, as they were found to be suitable for such tests over a two-week period.

Two groups of six animals were used in the experiment (12 in total). Another three animals were used for preliminary studies in order to establish detailed procedures and validate the overall research method. Each animal was induced with two difficult-to-heal wounds and provided with two variants of dressings: test and control materials. The control consisted of a commercial hydrogel wound dressing, i.e., a biomaterial with a structure similar to the tested material.

**Figure 1:**
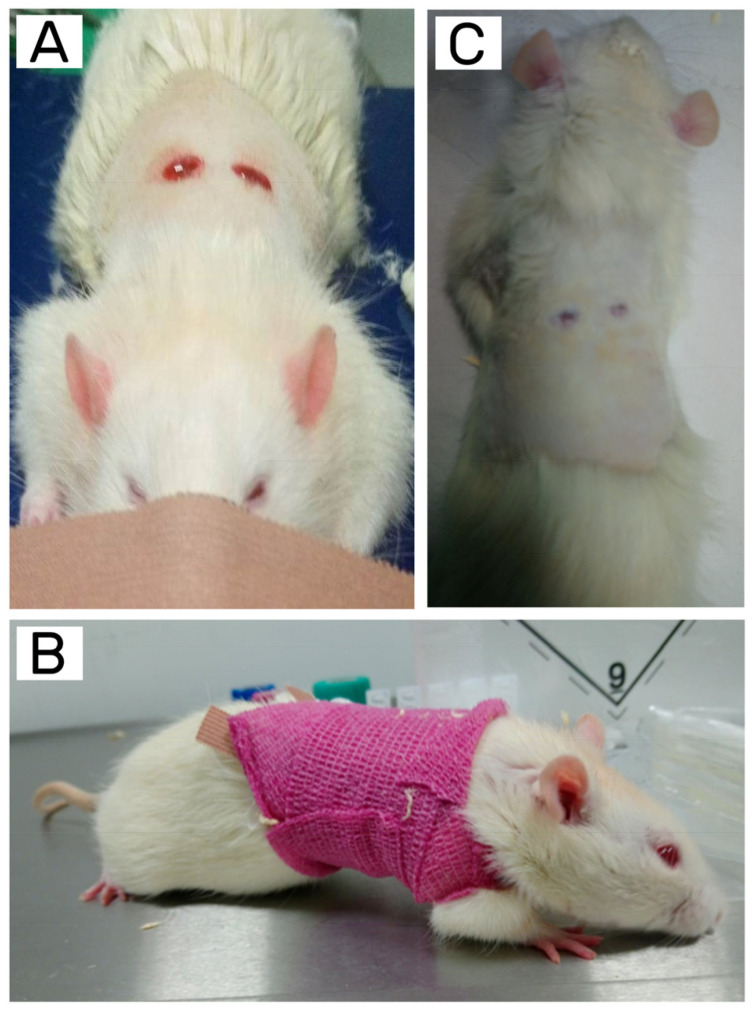
Animal model testing the effectiveness of the hydrogel dressings: (**A**) back with visible wounds immediately after the procedure; (**B**) rat during recovery with wounds protected by sterile compress, hypoallergenic plaster, and self-adhesive elastic bandage; (**C**) back of a rat after the seven-day wound healing process.

After placing the limbs on the operating table (with the back-up) and attaching the limbs to the table (thicker bands so as not to obstruct blood circulation), appropriate areas on the back were shaved and disinfected. The rats received the anesthetic isoflurane (5%) via inhalation during the procedure and the analgesic butorphanol (1–2 mg/kg body weight). Using surgical techniques, two nonpenetrating wounds (each approximately 1.5 cm in diameter) were created until bleeding occurred (Figure 1A). Then, a mitomycin C solution (1 mg/mL in phosphate-buffered saline) was applied to the wounds to suppress the natural proliferation of fibroblasts, keratinocytes, and epithelial cells. After 10 min, the wounds were thoroughly rinsed with a physiological saline solution, and the hydrogels, with a minimum diameter of 1.5 cm, were placed to cover each individual wound. The control hydrogel was placed on one side of the animal, and the hydrogel containing the test substance was placed on the other side. After placing the dressing on the skin defect, the whole wound was covered with a sterile compress, which was then fixed with a hypoallergenic plaster; finally, the whole body of the rat was wrapped with a self-adhesive elastic bandage (Figure 1B). Postoperative rats were allocated in individual sterile cages to avoid the mutual incursion of rats into healing zones and subjected to daily observation, which included weighing. The dressing was replaced every day, the wounds were exposed using surgical instruments, and the coverings were removed. As the hydrogel dressings, both the control and CMCS hydrogel, did not adhere to the wounds and, thus, did not damage the regenerating tissue, it was not necessary to anesthetize the animals with isoflurane when changing the dressings. The procedure was repeated for 7 days for the first group of animals and 14 days for the second. During the observation period, particular attention was paid to the rate of wound healing.

At the end of the observation period (i.e., 7 or 14 days), the rats were euthanized. The procedure was performed individually in isolation from other animals in the operating room. The animals were weighed and anesthetized by inhalation of isoflurane, then immobilized by gripping the skin of the neck and raised to expose the abdominal wall. The injection site was located in the lower right quarter of the rat abdomen. Injections in the left area should be avoided because of the possibility of colon damage. Sodium pentobarbital solution was administered intraperitoneally using a 22 G needle in a volume not exceeding 5 mL/kg body weight. After death was confirmed (no corneal reflexes, pupil dilation, and cessation of breathing and heart rate), the animal was placed on the operating table in the dorso-abdominal position (back side up—Figure 1C). Using microsurgical techniques, fragments of the healed tissues were dissected. The tissue samples were resected (24 skin sections from 12 animals) and subjected to macroscopic and microscopic evaluations to assess the tissue response to the tested materials. The test was carried out with the full blinding of the samples. Full-thickness skin sections were fixed in 4% buffered formalin, and the tissues were then macroscopically examined and dissected to obtain a defined wound area with a normal tissue margin. Subsequently, the tissues placed in the histological cassettes were subjected to standard paraffin treatment and cut on a microtome. Three sections were taken from every wound sample, each divided in two for one slide; thus, six sections from each individual wound were prepared for histopathological analyses. The obtained slides with sections were stained using the hematoxylin–eosin and van Gieson–Weighert methods. Based on the quality of the slides, three to five sections of the test tissues of every individual wound were selected for analysis.

Afterwards, the abdominal area was cut open. An autopsy examination was performed, including an examination of the body’s surface, natural orifices, chest, and abdominal cavity and its contents. The exposed organs were subjected to macroscopic examination to reveal possible vascular and tissue changes. Regional lymph nodes (axillary lymph nodes) were also assessed visually.

### 2.5. Statistical Analysis

The R programming language v4.3.1 (R Foundation for Statistical Computing, Vienna, Austria) within the RStudio environment v2023.06.1 (RStudio Inc., Boston, MA, USA) was employed to perform the statistical analysis. The amount of granulation tissue, inflammatory intensity or its nature, the direction of the collagen fibers, the collagen system, and the amount of young and mature collagen were compared between the control and test groups. The comparisons were made after one or two weeks of observation using Fisher’s exact test (“fisher.test” function in the “stats” package), with a *p*-value less than 0.05 considered as statistically significant. To control for multiple comparisons, a false discovery rate (FDR) < 0.25 was considered acceptable when performing Benjamini–Hochberg procedure via the “*p*.adjust” function in the “stats” package.

## 3. Results and Discussion

### 3.1. Carboxymethyl Chitosan Hydrogel Formed using the Radiation Technique

Polymers exposed to ionizing radiation may undergo a number of reactions induced mainly by free radicals, typically carbon-centered ones. These radicals, in most cases, cause degradation, crosslinking, atom transfer, or oxygen (or other additives, monomers, etc., if present) addition [27]. The first two processes directly influence the molecular weight of the polymer. Degradation, through main-chain scission, reduces the molecular weight of the polymer, whereas crosslinking, if involving radicals located on distinct macromolecules, doubles the molecular weight. Radiation processing may be conducted in a solid state or in solution; there may be other conditions suitable for inducing specific reactions, such as ‘in melt’ for thermoplastics. Hydrophilic or water-soluble polymers may be processed in aqueous solution in order to form hydrogels for biomedical applications. Commercially available solutions are based on synthetic hydrophilic polymers, such as poly(vinyl pyrrolidone), poly(ethylene glycol), or poly(vinyl alcohol). Their radiation processing forms durable hydrogels through crosslinking, while allowing for simultaneous sterilization [28]. This approach was taken in the current study based on a polymer of natural origin.

CMCS may be crosslinked using radiation in an aqueous solution, with the network’s formation taking place through the recombination of radicals located on the side methyl groups. These radicals are created by transient products of water radiolysis, chiefly hydroxyl radicals and hydrated electrons. In the case of simple polysaccharides, the OH radical is the main species that reacts with the polymer, whereas the hydrated electron has minor importance. However, the resulting CMCS hydrogels are only partially crosslinked, with a large soluble part (sol) remaining, and the material is weaker with a high swelling capability, which may prevent its use in foreseen applications [21,22]. The abovementioned properties are also a consequence of glycosidic linkages scission, which is always taking place with high yield in polysaccharides.

The application of a PEGDA crosslinker facilitates network formation, as this macromere has two unsaturated carbon–carbon bonds at both ends, allowing for OH radical addition and the formation of the carbon-centered radical. The hydrated electron also reacts with PEGDA, specifically with carbonyl function, resulting in the formation of a labile oxygen radical anion, which transforms to a carbon-centered radical [23,24]. Both reactions proceed at a high rate and high yield. Thus, PEGDA plays a crucial role in network formation through its radicals’ reactions with radicals on the polysaccharide, which result from either a direct reaction with OH or the degradation of the main chain due to the scission of glycosidic linkages. In the present context, it is more likely that PEGDA double bonds react with CMCS macroradicals to form intramolecular linkages. The hydrogels produced with the involvement of the PEGDA crosslinker are significantly stronger and with controlled swelling than those of the sole individual polysaccharides. Therefore, from the point of view of their handling properties, they seemed suitable for wound dressings and in vivo testing. In addition, the radiation treatment also sterilized the material for direct and safe application.

The animal experiments involved two difficult-to-heal wounds that were covered with the tested CMCS hydrogel and a control. The CMCS hydrogel samples were selected based on previous investigations of their liquid absorption potential, mechanical properties, integrity, and biocompatibility in vitro and in animals. These properties and their safety were tested beforehand with a series of CMCS hydrogels crosslinked with the assistance of a PEGDA macromer [10,24]. A gel formed using EB irradiation of 5% CMCS and 5% PEGDA solution with 25 kGy, ensuring effective sterilization, was selected because of its appropriate mechanical and biological properties (both in vitro and in animals), which have been demonstrated in a previous study [10]. The mechanical properties of the chosen gel were as follows: maximum compressive stress, δ, of 19.5 kPa; Young’s modulus of 29 kPa; and deformation at maximum compressive stress, ε, of 0.40 mm/mm. The hydrogel was soft, and its surface was neither tacky nor trickling; thus, its handling properties allowed for its application to wound.

Specific types of wound dressings are dedicated for using with different types of wounds; thus, hydrogels may be used on fresh wounds, burn wounds, or hard-to-heal wounds, as in the present case. Obviously, a comparator of a physical form similar to the tested hydrogels had to be used; thus, the control sample was qualitatively similar to the tested material: a commercial hydrogel dressing based on poly(vinyl pyrrolidone), crosslinked and sterilized using irradiation [28], available on the market under the trade name Aqua-Gel^®^. Both the control and test substances were hydrogels, but the control was based on a synthetic polymer network, with well-established properties and a long history of applications, while the tested hydrogel was based on a network of functionalized natural polymer of chitin: carboxymethyl chitosan.

### 3.2. Effectiveness of Wound Treatment with Hydrogel Dressings

Burn wounds are one of the most severe forms of skin damage, leading to prolonged hospitalization, long-term disability, or even death. Similar consequences may be caused by hard-to-heal wounds. The healing process is complicated and involves consecutive, overlapping, or parallel processes, such as inflammation, re-epithelialization, granulation, neovascularization, wound shrinkage, and further remodeling. The process can be significantly accelerated with the use of appropriate dressings, such as hydrogels, which are also often used because of their ability to cool, causing pain relief.

Laboratory rats were chosen as the animal model. Currently, there are no alternative methods for the histopathological assessment of angiogenesis and difficult-to-heal wound healing in vitro. Because of the anticipated use of the investigated hydrogel, in vivo tests may only be performed on vertebrates.

Observations of the animals over the course of the experiment showed no alarming symptoms, and their health was assessed as good. In the first days after the surgery, the rats were given an analgesic. The weight of the animals did not increase because of the relatively short observation period, post-operative discomfort, and restriction of movement by the dressing. During the daily change of the dressings, macroscopic observations of the wounds were also performed, which showed that the behavior of the CMCS hydrogels was good, similar to commercial gels. The tested hydrogel absorbed the wound exudate, while maintaining a moist wound healing environment. As anticipated, it was confirmed that the dressings did not cause inflammation, as previously demonstrated with cytotoxicity and biocompatibility tests. The tested and control hydrogel dressings, themselves, neither adhered to the wound nor grew into the regenerating tissue; therefore, it was undemanding and presumed painless to remove and change them. After the observation period, the tissues around the wounds were resected, and the animals were euthanized. The excised tissues were subjected to histopathological evaluation.

### 3.3. Histopathological Evaluation of the Effectiveness of the Hydrogel Dressings

A standard descriptive histopathological assessment of each skin section was performed [29,30,31,32,33,34,35] based on the following histological parameters: (1) amount of granulation tissue; (2) presence of inflammatory infiltrate; (3) direction of collagen fibers; (4) collagen system; (5) amount of young collagen; and (6) amount of mature collagen. In addition, the following were assed: (7) degree of skin formation; (8) disorders of differentiation and keratosis in young epidermis; (9) qualitative assessment of the inflammatory infiltration.

**Figure 2:**
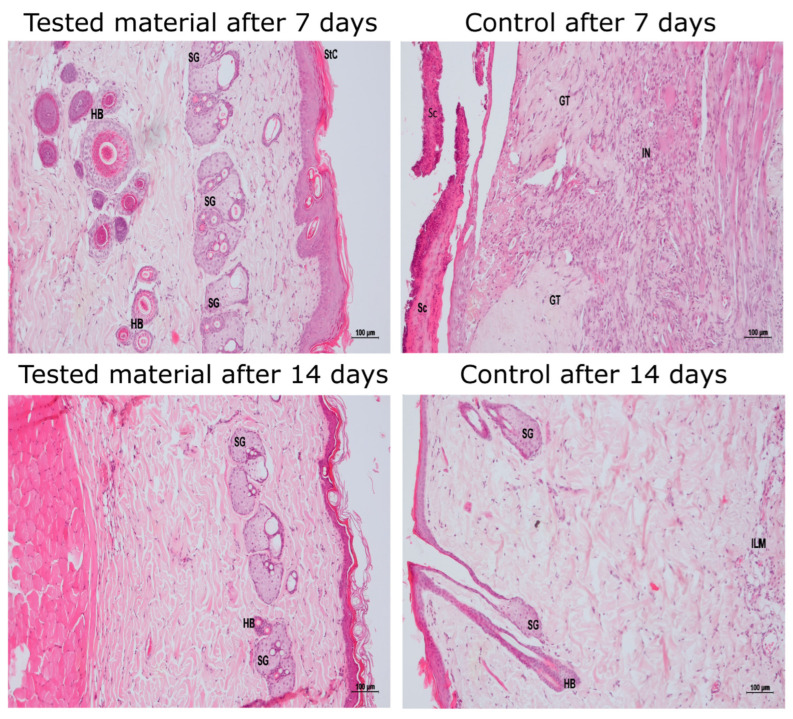
Histopathological images of wounds. Visualized are two samples of the CMCS and the control hydrogels, collected from one rat on day 7 and from another rat on day 14; HB—hair bulb; SG—sebaceous gland; StC—stratum corneum; Sc—scab; GT—granulation tissue; I—inflammation; L—lymphocytic; M—monocytic; N—neutrophilic. A comprehensive analysis of all wounds is presented in Supplementary Materials Table S1.

Exemplary histopathological images are provided in Figure 2, whereas a comprehensive analysis of all wounds is presented in Supplementary Materials Table S1. The images in Figure 2 show a comparison of the tested hydrogel and control materials after 7 and 14 days, which were collected from two rats, one rat for one period.

#### 3.3.1. Amount of Granulation Tissue

The amount of granulation tissue was determined according to the following classification: 1—abundant; 2—medium; 3—poor; and 4—lack. The occurrence of granulation was subjected to statistical analysis (Table 1).

The tested hydrogel demonstrated significantly less granulation tissue in the wounds after the first period than the controls. Small amounts of granulation tissue were noted in the first week for the CMCS hydrogel; while this generally hinders wound healing, this also indicates that healing is proceeding properly. In both the control and test groups, the healing increased to poor in the second week, indicating a lack of significant pathological process. This may result from the mechanical action of the irritating factor, which also caused connective tissue remodeling. Increasing the amount of granulation tissue in the later stage of wound healing may also have positive effects on the healing process; it was found to be related with a smoother surface and less scarring, which was also noted previously [36,37,38,39,40].

No statistically significant difference (but with a trend that approached significance) in the amount of granulation tissue was observed between the two groups after seven days (*p* < 0.05). The CMCS hydrogel dressing was more likely to demonstrate poor granulation than the control (fraction of 0.67 vs. 0.0). While the control was typically abundant, the studied material did not demonstrate abundant or average granulation (fraction of 0.50 vs. 0.0). The structure similarity index, which indicates difference between two groups, was only 33%.

After 14 days, no statistically significant difference was found between the two groups (*p* > 0.05); however, the structure similarity index was much higher (83%). No statistically significant difference was found between the one-week and two-week measurements in the study group (*p* = 0.1775; FDR > 0.25).

#### 3.3.2. Intensity of Inflammatory Infiltration

The intensity of the inflammatory infiltration was defined as follows: 1—abundant; 2—moderate; 3—poor; and 4—lack (Table 2).

The presence of inflammatory infiltrate, especially at high levels and for long durations, is an unfavorable factor. The tested material appeared to have a very beneficial effect, demonstrating slight inflammatory infiltrate compared to a moderate level for the control wounds. Importantly, minor inflammation infiltration may facilitate the removal of necrotic tissue, kill local bacteria, and promote wound healing [41,42].

A statistically significant difference in the intensity of the inflammatory infiltration was observed in the material tested after 7 days compared to the control (*p* > 0.05). However, the wound treated with the CMCS hydrogel typically showed no infiltration significantly more often than in the control (fraction of 0.67 vs. 0.0). In addition, the infiltration was most often abundant in the control compared to no profuse infiltration in the tested material (fraction of 0.67 vs. 0.0). The structure similarity index (16%) confirms the existence of some difference between the tested and control materials.

However, after 14 days, no statistically significant difference in the intensity of inflammatory infiltration was found between the groups (*p* > 0.05). However, the structure similarity index was much higher (66%). Moderate, sparse, and no infiltration (fractions of 0.33) were present with the same frequency in the two groups, and moderate infiltration (fraction of 0.67) dominated in the control.

In the study group, no significant difference was found between the severity of the inflammatory infiltration observed after one week and after two weeks (*p* = 0.7403; FDR > 0.25), suggesting that significant wound healing occurred after a period of one week. As such, there was no need to use the tested hydrogel dressings for a long time, because the wound was no longer a difficult wound.

#### 3.3.3. Direction of Collagen Fibers

The direction of the collagen fibers was assessed as follows: 1—vertical; 2—mixed; and 3—horizontal (Table 3).

Typically, mature collagen fibers tend to have a horizontal course and rarely a vertical one. In many cases, the course is mixed, indicative of the gradual maturation of young connective tissue. In wounds with the tested material, the fibers quickly developed a horizontal direction compared to the control, which is a favorable result. This was superseded by a mixed system at the second week, like the control, which may indicate an intensification of the collagen fibers’ remodeling and their adaptation to accommodate the loads affecting the skin [43,44,45].

No significant difference was found in the direction of the collagen fibers between the material tested after seven days compared to the control (*p* > 0.05). However, some differences should be noted. In the CMCS hydrogel dressing, the direction of the collagen fibers was mostly horizontal (fraction of 0.67) and less often mixed (fraction of 0.33). In contrast, in the controls, the direction of the collagen fibers was very evenly distributed, with vertical, mixed, and horizontal directions (fractions of 0.33 each) occurring with the same frequency. The structure similarity index (66%) confirms a relatively small difference in the structures between the tested and control materials.

Similarly, no statistically significant difference was found after 14 days in the direction of the collagen fibers between the two groups (*p* > 0.05). The tested material presented collagen fibers in all directions at slightly different frequencies; the fractions were 0.17 for vertical, 0.50 for mixed, and 0.33 for horizontal. However, in the control after 14 days, only the mixed direction was present (fraction of 1.0). The structure similarity index was 50%, which confirms that the difference in the structures between the groups was quite significant but not statistically so.

No significant difference in the course of the collagen fibers was found in the study group between one week and two weeks (*p* = 0.5671; FDR > 0.25).

#### 3.3.4. Collagen System

The collagen system was defined as: 1—reticular; 2—mixed; and 3—bundle (Table 4).

The results are very similar to the course of the collagen fibers. Regular, mature collagen commonly has a bundle arrangement, with a reticular arrangement being the least favorable. A mixed configuration indicates the gradual maturation of young connective tissue. In wounds with the CMCS hydrogel dressing, the fibers quickly obtained a mixed/bundle pattern compared to the control, which is a favorable result. Nevertheless, at the second week, the wounds with the tested material displayed a mixed system, similar to the control; this may indicate more intense collagen fiber remodeling and the adjustment of their number and structure to accommodate the loads affecting the skin [39,40].

No statistically significant difference in the collagen system was found between the groups after seven days (*p* > 0.05). However, in the CMCS hydrogel dressing, collagen bundles were the most common type (fraction of 0.67), much more so than in the controls (fraction of 0.17). In addition, the reticular collagen system was more common in the control (fraction of 0.67; most common) than in the test material (fraction of 0.17). The mixed system was observed at the same frequency in both groups (fractions of 0.17). The structure similarity index (50%) confirms that some difference in the structures existed between the tested material and the control.

Similarly, after 14 days, no statistically significant difference was found in the collagen system between the CMCS hydrogel dressing and the control materials (*p* > 0.05). The structure similarity index was much higher (83%), indicating a high similarity between the structures. The collagen composition in both of the compared materials was mostly mixed (fractions amounting to 0.67), and in the tested material, a collagen system was observed more often than in the control (fractions of 0.33 and 0.17). No significant differences in the collagen system of the study group were observed between one week and two weeks (*p* = 0.2424; FDR > 0.25).

#### 3.3.5. Amount of Young Collagen

The amount of young collagen was assessed according to the following: 1—abundant; 2—moderate; 3—minimal; and 4—lack (Table 5).

A large amount and long persistence of young collagen are disadvantageous factors, which inhibit maturation and crosslinking. A lower amount of young collagen was noted in wounds with the CMCS hydrogel dressing, which is a better prognostic factor compared with the control [46,47,48,49].

No statistically significant difference was noted in the amount of young collagen observed in the two groups after 7 days (*p* > 0.05). However, the amount of young collagen in the CMCS hydrogel dressing was most often minimal or even absent (fractions of 0.33 each), while abundant (fraction of 0.50) or moderate amounts (fraction of 0.33) were noted in the control. The structure similarity index (50%) confirms that a moderate difference in the structures existed between the tested material and the control.

No statistically significant differences were found between the two groups after 14 days (*p* > 0.05). The material most often contained a minimal amount of young collagen (fraction of 0.67) and some moderate amounts (fraction of 0.33). However, in the control, the highest amount of moderate collagen (fraction of 0.67) and minimal amount of young collagen (fraction of 0.33) were observed after 14 days. The structure similarity index was 66%, indicating a small but statistically insignificant difference between the groups. In addition, no significant difference was observed between the groups after one week (*p* = 0.4156; FDR > 0.25).

#### 3.3.6. Amount of Mature Collagen

The amount of mature collagen was assessed as follows: 1—abundant; 2—moderate; 3—minimal; and 4—lack (Table 6).

A large amount of mature collagen is a beneficial factor and indicates proper maturation. Indeed, the collagen under the CMCS hydrogel dressing was found to mature faster and undergo crosslinking in the early stage of wound healing. Following this, the amount of collagen decreased to the control’s levels; this may be a consequence of the remodeling of the collagen fibers and the adjustment of their number and structure to the size and strength of the loads acting on the skin. In addition, only small amounts of young collagen were noted under the tested material, indicating proper maturation of connective tissue. In contrast, young collagen persisted for a longer period in the control samples [36,46,47,48,49,50,51,52].

After seven days, the amount of mature collagen differed significantly between the test group and the controls (*p* < 0.05). The amount of mature collagen was mostly abundant (fraction of 0.83) in the wound treated with the CMCS hydrogel. In contrast, in the controls, most of the samples demonstrated minimal (fraction of 0.50) or moderate levels (fraction of 0.33) of mature collagen. The structure similarity index (33%) indicates a large structural difference between the tested and control materials.

After 14 days, most of the CMCS hydrogel dressings demonstrated a moderate amount of mature collagen (fraction of 0.67), followed by abundant (fraction of 0.17) and minimal levels (fraction of 0.17). No significant difference was observed in the control material (*p* = 1.0), and the two groups demonstrated a very high structural similarity (100%). Likewise, no significant difference was observed between one and two weeks in the test group (*p* = 0.0801; FDR > 0.25).

#### 3.3.7. Degree of Skin Formation

The degree of skin formation was assessed according to the following classification: 1—lack; 2—short epidermis protrusions; 3—long epidermal bridges; and 4—full coverage of the wound with epidermis. A statistical comparison of the skin formation is shown in Table 7.

The CMCS hydrogel dressing was found to promote the regeneration of the epidermis during healing, especially in the early stage, compared with the controls [53,54,55]. After seven days, no significant difference (but suggestive trend of significance) in the degree of skin formation was found between the test and control groups (*p* > 0.05). In all cases of the CMCS hydrogel dressings, full epidermal coverage of the wound was observed (fraction of 1.0). In the controls, full coverage, short protrusions, and no epidermis were found in equal amounts (fraction of 0.33). The structure similarity index (33%) confirms that large differences existed between the tested and the control materials, with the advantage belonging to the tested material.

After 14 days, the CMCS hydrogel dressing typically showed full coverage of the wound with epidermis (fraction of 0.67) or none (fraction of 0.33). In the controls, full coverage of the wound was most commonly observed (fraction of 0.67), followed by epidermal protrusions (fraction of 0.33). No statistically significant difference was noted between the two groups in this regard (*p* > 0.05), indicated also by the structure similarity index (67%). No significant difference in epithelium cover was noted between one week and two weeks in the test group (*p* = 0.4545; FDR > 0.25), with a structure similarity index of 71.0%. Hence, the structures in the tested materials after 7 and 14 days were quite similar.

#### 3.3.8. Disorders in Differentiation and Keratosis in the Young Epidermis

Disorders in differentiation and keratosis in the young epidermis were defined according to the following classification: 1—plentiful; 2—moderate; 3—significant; and 4—minimal. A statistical comparison of the parameters of the young epidermis is shown in Table 8.

In the first stage of healing, the young epidermis developed more correctly under the CMCS hydrogel dressing than the control, as indicated by the presence of the minimum amount of differentiation disorders and keratinization. However, in week two, the condition of the young epidermis under the tested material became comparable to the control. This may be a consequence of the irritating mechanical effect of the dressing itself or of the contamination of the wound and superinfection [56,57].

After seven days, no significant differences (but suggestive of a trend of significance) in the occurrence of differentiation and keratosis disorders were found between the test material and the controls (*p* < 0.05). In the CMCS hydrogel dressings, minimal disturbances were typically observed (fraction of 0.83), with only one moderate disturbance (fraction of 0.17). In the controls, profuse disturbances were frequently observed (fraction of 0.67), with two cases of minimal disturbances (fraction of 0.33). The structure similarity index (33%) confirms a considerable difference between the tested material and the control, with the advantage of the tested material.

After 14 days, a significant difference in the occurrence of differentiation and keratosis disorders was found between the test material and the controls (*p* < 0.05). In the CMCS hydrogel dressing, minimal disturbances were most often observed (fraction of 0.50), followed by profuse (fraction of 0.33) or moderate disturbances (fractions of 0.17). In the control group, moderate disturbances were most often observed (fraction of 0.67), followed by significant changes (fraction of 0.33). The structure similarity index (17%) confirms large structural differences existed between the two groups. No significant differences in the frequency of differentiation and keratosis disorders were found between one and two weeks in the study group (*p* = 0.6970; FDR > 0.25).

#### 3.3.9. Qualitative Nature of the Inflammatory Infiltrate

The qualitative nature of the inflammatory infiltrate was classified according to the following: N—neutrophilic (purulent); L—lymphocytic; L/M—lymphocytic–histiocytic; N/L/M—mixed; and X—lack. A statistical comparison is shown in Table 9.

The process of wound healing is characterized by the following stages: (a) neutrophilic (i.e., purulent) infiltration—cells clean up necrotic tissues and infectious pathogens by phagocytosis (i.e., acute inflammation); (b) lymphocytic infiltration, which determines the mechanisms of cytotoxicity-dependent immunity; (c) histiocytic infiltration, which removes dead tissue, damaged collagen fibers, and attenuated neutrophils, and this most often coexists with lymphocytic infiltration. Histiocytes are also often involved in connective tissue remodeling. Of these, the least favorable is profuse, long-lasting neutrophilic (purulent) infiltration: any long-lasting inflammatory process at the wound site hinders healing. In the described experiment, wounds with the CMCS hydrogel dressing showed a shortened phase of neutrophilic infiltration and persistence of a weak infiltration, mainly of a mixed nature. This indicates that the inflammatory process is being reduced quite quickly [58,59].

After seven days, no significant (but near-significant trend) difference was found between the groups with regard to the composition of the inflammatory infiltrate (*p* > 0.05). The CMCS hydrogel dressing demonstrated ‘no infiltration’ (0.67) significantly more frequently than the controls (0.0). In the controls, the infiltration was most commonly neutrophilic (purulent) (0.67), significantly more so than in the tested material (0.17). The similarity index regarding the composition of the infiltration between the tested material and the controls (33%) indicates a substantial yet statistically insignificant difference.

Similarly, after 14 days, no statistically significant difference in the composition of the inflammatory infiltrate was found between the groups (*p* > 0.05). The structure similarity index was higher (66%). The test groups demonstrated lymphocytic–histiocytic infiltration and no infiltration in equal amounts (fractions of 0.33); the controls were dominated to a greater degree by lymphocytic–histiocytic infiltration (fraction of 0.50). No significant difference in the composition was observed between one and two weeks in the test group (*p* = 0.6104; FDR > 0.25).

#### 3.3.10. General Evaluation of the Histopathological Results

The general results of the histopathological examinations can be summarized as follows. Wounds with the CMCS hydrogel dressing showed better parameters of skin wound healing compared with the commercial hydrogel dressing, especially in the first week. A slowdown in wound healing was noted in week 2; this could be a consequence of the dressing having a slight irritating effect, causing the animal to gain interest in the dressing or even overweighting the young wound (least likely). Despite the slowing wound healing and some remodeling of the young connective tissue in the CMCS hydrogel group, the condition of the wound was still better than in the control, especially in terms of the amount of inflammatory infiltrate, the amount of young collagen, and the epithelialization degree. The arrangement and direction of the collagen fibers were also somewhat better than in the control. Moreover, the rate of skin coverage was good, and its remodeling was effective upon application of the CMCS hydrogel dressing. It is worth noting that the animals used to prepare the method were also subjected to brief macroscopic and histopathological examinations. Wounds without any treatment healed worse compared to both the tested and control hydrogels, which validated the wound and the control material.

Our findings indicate the presence of a regular healing pathway, similar to those reported for advanced wound management strategies. The new CMCS hydrogel dressings represent significant potential in positively influencing wound healing parameters, such as the amount of inflammatory infiltration or young collagen, or the degree of epithelialization, with inherently very low or zero cytotoxicity.

It is known that chitosan-containing dressings induce wound covers with uniformly fibrous conformations with a large surface area, which enables oxygen penetration and fluid exchange, as well as maintaining an appropriate environment at the interface between the wound and the dressing, as in the case of native chitosan [15]. Similarly, the CMCS dressings used in the present study absorbed wound exudate while maintaining a moist wound healing environment thanks to the hydrogel structure (i.e., the embedded water) and the nature of the polymer itself, i.e., the retained biological properties of chitosan. Moreover, the dressings did not adhere to the wound or grow into the regenerating tissue. The literature shows that chitosan dressings inhibit the growth of bacteria [60] and can prevent the development of pathogenic species that can initiate wound infection [15]. The current results confirm that the dressings did not cause inflammation, which was also demonstrated earlier through cytotoxicity and biocompatibility studies. The CMCS hydrogels generate positive trends in healing, similar to those observed for effective dressings [55,58,61,62,63], but distinct to the examples in the literature, the current hydrogel material was synthesized and sterilized with radiation.

## 4. Conclusions

Carboxymethyl chitosan-based hydrogels manufactured using ionizing radiation appear to possess beneficial wound healing properties. The method of synthesis and the final composition of the hydrogel (i.e., polysaccharide and crosslinker concentrations) were chosen based on the evidence that irradiation induces crosslinking of CMCS. The addition of PEGDA to the CMCS aqueous solutions results in the formation of durable macroscopic gels, even at relatively low radiation doses of a few kGy. Moreover, at an applied dose of 25 kGy or more, as in the present case, the gel is sterilized while also being synthesized.

In in vivo experiments of hard-to-heal wound treatment, no adverse symptoms were observed in the animals, and their health was assessed as good during the course of healing. During the daily change of the dressings, macroscopic observations of the wounds showed that the effectiveness of the CMCS hydrogel was similar to that of the commercial gel dressing. The CMCS hydrogel absorbed the wound exudate, maintained a moist wound environment and, in general, performed appropriately. The hydrogel positively influenced the wound healing; particularly, the results of the amount of inflammatory infiltration, young collagen, and the degree of epidermalization were improved. Moreover, the performance of bio-based hydrogels in wound healing support is not deficient in comparison to a commercial solution, but the natural platform of the network should be regarded as being valuable. Despite the intended application of hydrogels for the treatment of recalcitrant wounds, such as burns, they may also be suitable as drug carriers. Our optimistic results suggest that after scaling and optimization of the production technology, prototypes of such hydrogel systems may be available for certification. The use of the proposed dressings may shorten treatment times for recalcitrant wounds, thus improving quality of life and reducing the costs of medical care.

## Figures and Tables

**Table 1:** Granulation tissue in the control group and in the tested material after one and two weeks of observation.

Amount of Granulation Tissue	One Week			
	Control Hydrogel		CMCS Hydrogel	
	N	Fraction	N	Fraction
Abundant	3	0.50	-	-
Mean	1	0.17	-	-
Poor	-	-	4	0.67
Absent	2	0.33	2	0.33
Comparison of the tested material with the control material	Fisher’s exact test, *p* = 0.0563 (FDR < 0.25)			
	**Two Weeks**			
Abundant	1	0.17	1	0.17
Mean	1	0.17	2	0.33
Poor	2	0.33	1	0.17
Absent	2	0.33	2	0.33
Comparison of the tested material with the control material	Fisher’s exact test, *p* = 1.0000 (FDR > 0.25)			

**Table 2:** Inflammatory infiltration (intensity) in the control group and in the study materials after one and two weeks.

Inflammatory Infiltration (Intensification)	One Week			
	Control Hydrogel		CMCS Hydrogel	
	N	Fraction	N	Fraction
Abundant	4	0.67	-	-
Moderate	-	-	1	0.17
Poor	2	0.33	1	0.17
Absent	-	-	4	0.67
Comparison of the tested material with the control material	Fisher’s exact test, *p* = 0.0129 (FDR < 0.25)			
	**Two Weeks**			
Abundant	-	-	1	0.17
Moderate	4	0.67	1	0.17
Poor	2	0.33	2	0.33
Absent	-	-	2	0.33
Comparison of the tested material with the control material	Fisher’s exact test, *p* = 0.1775 (FDR > 0.25)			

**Table 3:** Direction of the collagen fibers in the control group and in the tested material after 1 and 2 weeks of the study.

Direction of Collagen Fibers	One Week			
	Control Hydrogel		CMCS Hydrogel	
	N	Fraction	N	Fraction
Vertical	2	0.33	-	-
Mixed	2	0.33	2	0.33
Horizontal	2	0.33	4	0.67
Comparison of the tested material with the control material	Fisher’s Exact test, *p* = 0.4805 (FDR > 0.25)			
	**Two Weeks**			
Vertical	-	-	1	0.17
Mixed	6	1.0	3	0.50
Horizontal	-	-	2	0.33
Comparison of the tested material with the control material	Fisher’s exact test, *p* = 0.1818 (FDR > 0.25)			

**Table 4:** Collagen system in the control group and in the tested material after 1 and 2 weeks of the study.

Collagen System	One Week			
	Control Hydrogel		CMCS Hydrogel	
	N	Fraction	N	Fraction
Reticulate	4	0.67	1	0.17
Mixed	1	0.17	1	0.17
Bundle	1	0.17	4	0.67
Comparison of the tested material with the control material	Fisher’s exact test, *p* = 0.3506 (FDR > 0.25)			
	**Two Weeks**			
Reticulate	1	0.17	-	-
Mixed	4	0.67	4	0.67
Bundle	1	0.17	2	0.33
Comparison of the tested material with the control material	Fisher’s exact test, *p* = 1.0000 (FDR > 0.25)			

**Table 5:** The amount of young collagen after 1 and 2 weeks.

Amount of Young Collagen	One Week			
	Control Hydrogel		CMCS Hydrogel	
	N	Fraction	N	Fraction
Abundant	3	0.50	1	0.17
Moderate	2	0.33	1	0.17
Minimal	1	0.17	2	0.33
Absent	-	-	2	0.33
Comparison of the tested material with the control material	Fisher’s exact test, *p* = 0.4935 (FDR > 0.25)			
	**Two Weeks**			
Abundant	-	-	-	-
Moderate	4	0.67	2	0.33
Minimal	2	0.33	4	0.67
Absent	-	-	-	-
Comparison of the tested material with the control material	Fisher’s exact test, *p* = 0.5671 (FDR > 0.25)			

**Table 6:** Amount of mature collagen in the control group and in the tested material after 1 and 2 weeks of the study.

Amount of Mature Collagen	One Week			
	Control Hydrogel		CMCS Hydrogel	
	N	Fraction	N	Fraction
Abundant	1	0.17	5	0.83
Moderate	2	0.33	1	0.17
Minimal	3	0.50	-	-
Abstract	-	-	-	-
Comparison of the tested material with the control material	Fisher’s exact test, *p* = 0.0801 (FDR < 0.25)			
	**Two Weeks**			
Abundant	1	0.17	1	0.17
Moderate	4	0.67	4	0.67
Minimal	1	0.17	1	0.17
Abstract	-	-	-	-
Comparison of the tested material with the control material	Fisher’s exact test, *p* = 1.0000 (FDR > 0.25)			

**Table 7:** Degree of skin formation in the control group and in the tested material after 1 and 2 weeks.

Degree of Skin Formation	One Week			
	Control Hydrogel		CMCS Hydrogel	
	N	Fraction	N	Fraction
Lack	2	0.33	-	-
Short cuticle protrusions	2	0.33	-	-
Long epidermal bridges	-	-	-	-
Full coverage of the wound with epidermis	2	0.33	6	1.0
Comparison of the tested material with the control material	Fisher’s exact test, *p* = 0.0606 (FDR < 0.25)			
	**Two Weeks**			
Lack	-	-	2	0.33
Short cuticle protrusions	2	0.33	-	-
Long epidermal bridges	-	-	-	-
Full coverage of the wound with epidermis	4	0.67	4	0.67
Comparison of the tested material with the control material	Fisher’s exact test, *p* = 0.2121 (FDR > 0.25)			

**Table 8:** Disorders in differentiation and keratosis in the young epidermis in the control group and in the tested materials after 1 and 2 weeks of the study.

Differentiation and Keratosis Disorders	One Week			
	Control Hydrogel		CMCS Hydrogel	
	N	Fraction	N	Fraction
Plentiful	4	0.67	-	-
Moderate	-	-	1	0.17
Significant	-	-	-	-
Minimal	2	0.33	5	0.83
Comparison of the tested material with the control material	Fisher’s exact test, *p* = 0.0606 (FDR < 0.25)			
	**Two Weeks**			
Plentiful	-	-	2	0.33
Moderate	4	0.67	1	0.17
Significant	2	0.33	-	-
Minimal	-	-	3	0.50
Comparison of the tested material with the control material	Fisher’s exact test, *p* = 0.0368 (FDR < 0.25)			

**Table 9:** Qualitative nature of the inflammatory infiltrate in the control group and in the tested material after 1 and 2 weeks.

Inflammatory Infiltrate (Composition)	One Week			
	Control Hydrogel		CMCS Hydrogel	
	N	Fraction	N	Fraction
Neutrophilic	4	0.67	1	0.17
Lymphocytic	1	0.17	-	-
Lymphocytic–histiocytic	1	0.17	1	0.17
Mixed	-	-	-	-
Lack	-	-	4	0.67
Comparison of the tested material with the control material	Fisher’s exact test, *p* = 0.0693 (FDR < 0.25)			
	**Two Weeks**			
Neutrophilic	-	-	-	-
Lymphocytic	1	0.17	1	0.17
Lymphocytic–histiocytic	3	0.50	2	0.33
Mixed	2	0.33	1	0.17
Lack	-	-	2	0.33
Comparison of the tested material with the control material	Fisher’s exact test, *p* = 0.7403 (FDR > 0.25)			

## Data Availability

The data presented in this study are available upon request from the corresponding authors.

## Supplementary Materials

The following supporting information can be downloaded at: https://www.mdpi.com/article/10.3390/jfb14090473/s1. Table S1: Images and histopathological evaluation of wound samples from rat material.
