# Supplementary material for: Carboxymethyl Chitosan Hydrogels for Effective Wound Healing—An Animal Study
Source: J Funct Biomater. 2023 Sep 15;14(9):473. doi: 10.3390/jfb14090473 (PMC10531907; doi:10.3390/jfb14090473)
Supplement: Supplementary file 1 [file jfb-14-00473-s001.zip › jfb-2538745-supplementary.pdf]

<table>
<tr><th rowspan="2">Time</th><th rowspan="2">ID of rats</th><th colspan="2">Images and histopathological evaluation of wound samples from rat material</th></tr>
<tr><th>P – Control material</th><th>L - Tested material</th></tr>
<tr><td rowspan="2">One week</td><td rowspan="2">1</td><td>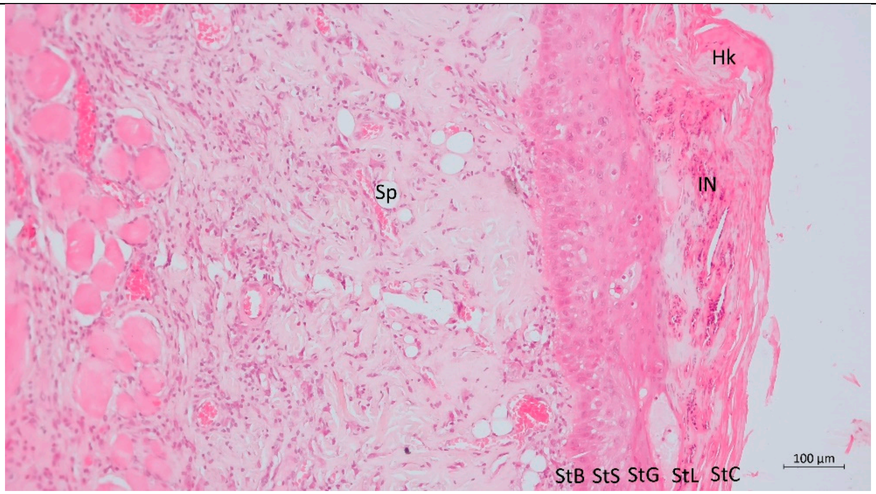
</td><td>no data available</td></tr>
<tr><td>Visible weak-moderate growth of the basal layer with signs of cell activation and rejuvenation and a significant increase in the thickness of the spinous layer compared to normal skin in the wound area. The granular layer of the epidermis does not deviate from the norm. The stratum corneum is mostly normal, but foci of thickening are found, suggesting hyperkeratosis. The foci of hyperkeratosis correspond to the following visible in the deeper layers: swelling of the spinous layer with the formation of cysts filled with exudative fluid - epidermal spongiosis. In foci of hyperkeratosis, exudate and edema are also found, which coagulate to form a serous scab containing numerous neutrophils and single macrophages. The picture described indicates an advanced healing process with closed epidermis. In the young epidermis, a probable bacterial superinfection is visible and, as a consequence, serous-purulent changes are observed.</td><td>The histopathological picture is similar to that of the 1P rat, however, the changes in the epidermis and the delayed remodeling process of the dermis are much more intense. Much more intense are keratinization and inflammatory neutrophilic infiltration with a highly purulent picture combined with abundant, coagulating exudate on the surface of the young epidermis. Within the clot, cellular detritus and foci resembling basophilic bacteria are visible, mixed with inflammatory infiltrate and cellular aggregate.</td></tr>
</table>

| | | |
|---|---|---|
| 2 | 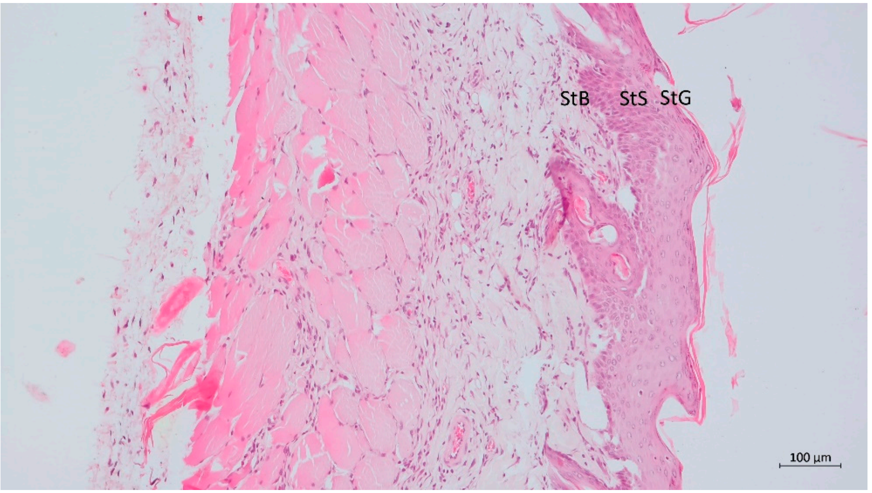 | 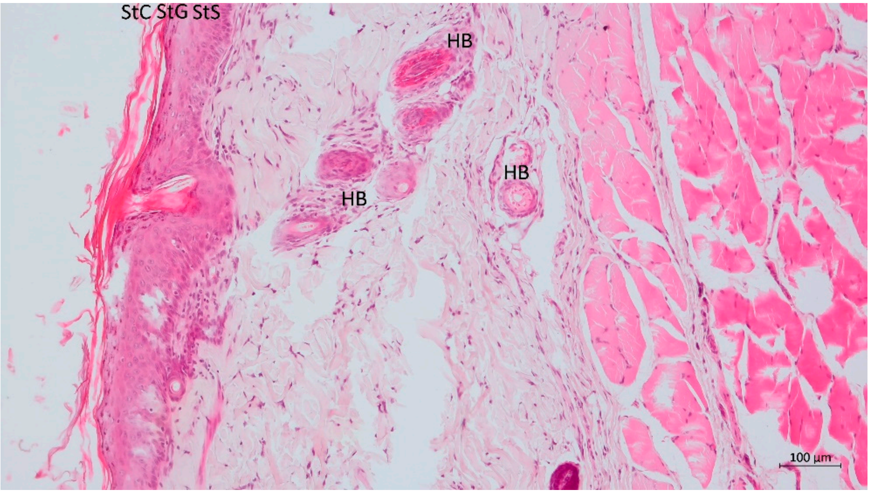 |
| | Visible young epidermis, not containing hair, and young connective tissue of the dermis, which is a healed wound in the remodeling phase. The epidermis within the healing wound is thickened with a particular widening of the spinous layer and the focal granular layer. The keratinizing layer is relatively thin. The inflammatory infiltrate is poor, lymphocytes predominate, macrophages and neutrophils are less numerous. | Difficulty in identifying the healing focus, because the entire skin in the obtained preparations looks similar. Only a certain discrepancy from the norm and the surrounding skin shows a focus of thickening of the spinous and granular layer (with numerous cytoplasmic granules) and some features of rejuvenation of the epidermis. Hair follicles appear in the observed fragment of the skin. No visible inflammatory infiltration and rejuvenation of the connective tissue of the dermis. This indicates a very advanced healing process - more intense compared to the control. |
| 3 | 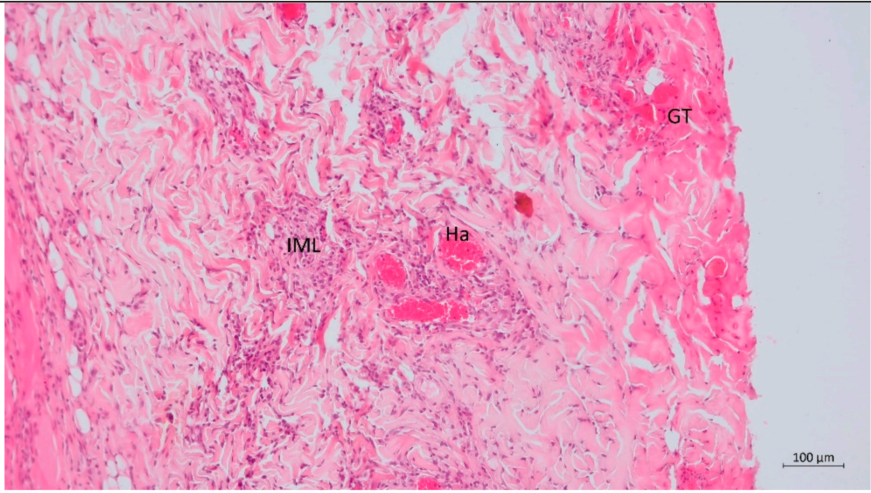 | 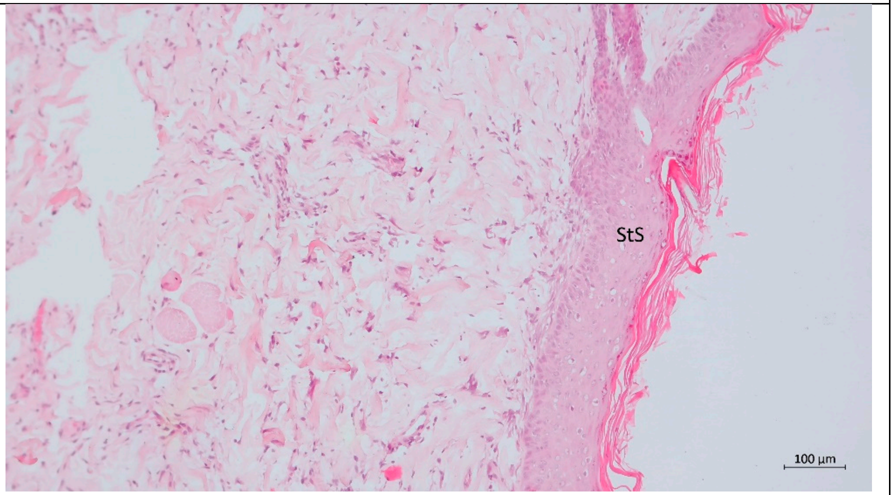 |

| | | | |
|---|---|---|---|
| | | The wound focus is easy to identify due to the very poor epidermis. Only short epidermal projections are visible. Granulation tissue and activated fibroblasts and a rich inflammatory infiltrate in the skin are visible. The infiltrate is dominated by lymphocytes and monocytes. The dermis is dominated by young collagen with a chaotic, mixed course that does not form bundles. Visible dilated vessels, filled with blood in the dermis. | The wound focus is covered with fully young epidermis with a slight expansion of the spinous layer with normal keratosis. No visible inflammatory infiltrate. Collagen in the form of bundles with a mixed course. The process of angiogenesis is also completed. Single clusters of young epithelial cells are visible - probably the buds of new hair follicles. |
| | 8 | 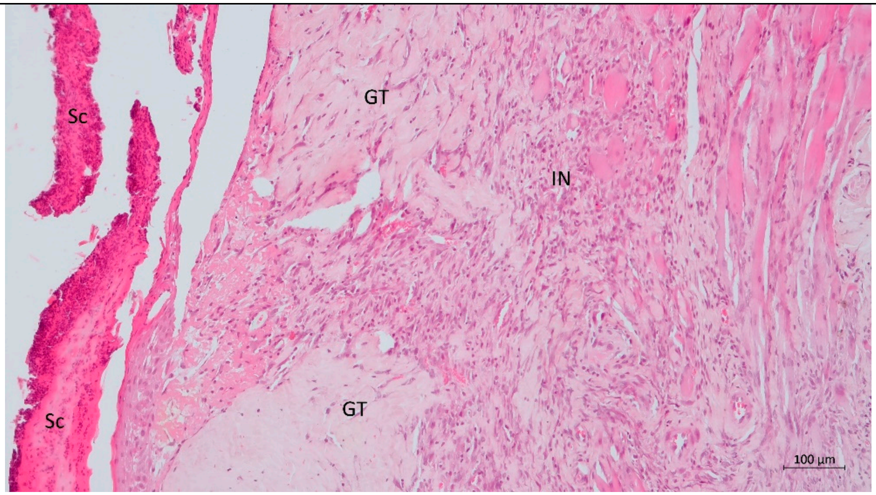  | 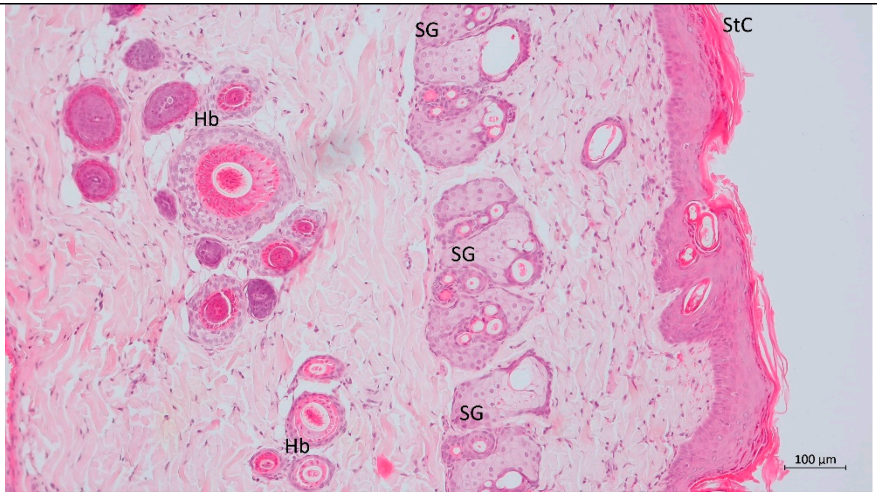  |
| | | Wound easily identifiable. Visible short epidermal tongues. The areas of epidermis and the wound not covered with epidermis are covered with a clot of serous secretion, in which rich cells of inflammatory infiltration are present, composed of neutrophils - of a purulent nature. Between the layer of the dermis and the clot/young epidermis, a homogenous, eosinophilic mass with a fibrin structure is visible - probably an organizing serous clot. In the dermis, foci of granulation tissue with numerous fibroblasts and a mixed infiltrate with dominant neutrophils and histiocytes are visible. No apparent angiogenesis. The described process may indicate bacterial overgrowth of the wound and hindering the healing process. | There are no distinct differences between different skin areas. Only a less regular arrangement of collagen and a small number of hair follicles and sebaceous glands with significant cell rejuvenation are visible. Above the described area, the epidermis has a clearly expanded granular layer. Only these changes may indicate a complete healing of the wound. |

<table>
<tr><td rowspan="2">9</td><td>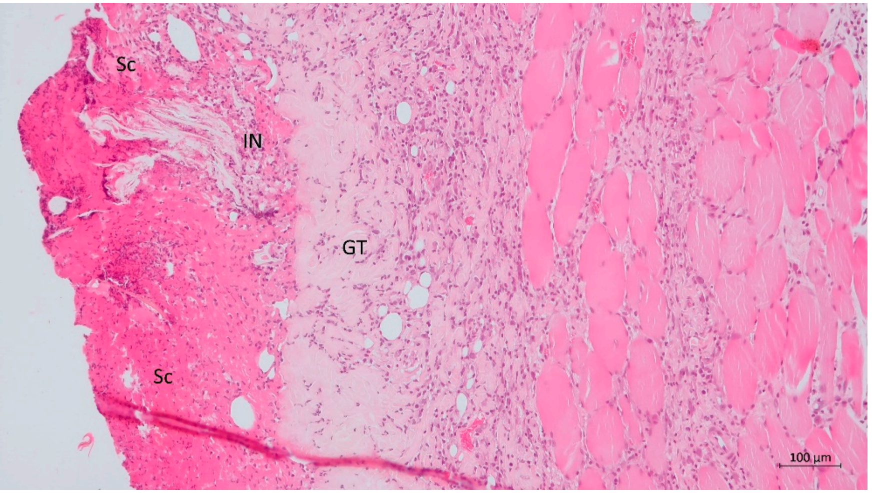
</td><td>no data available</td></tr>
<tr><td>No visible epithelial features. A rich-cell serous clot adheres to the edge of the wound with the epidermis, covering the entire wound. The clot is dominated by inflammatory cells - neutrophils. In the clot itself, cavities filled with eosinophilic, homogeneous exudate are visible. A very rich granulation tissue is visible, especially at the edges of the wound. The wound center is dominated by a mixed infiltrate dominated by lymphocytes and histiocytes, which covers the dermis, subcutaneous tissue and muscles. Lack of any signs of healing, probably due to bacterial superinfection.</td><td>The wound is easy to identify. Advanced repair and healing processes. The entire wound surface is covered with young epidermis. In the epidermis, the following layers are visible: basal, spinous and granular. Nevertheless, the keratinization process is proceeding properly. The dermis is dominated by a horizontal collagen bundle system. Within the dermis - the reticular layer and muscles, a weak lymphocytic-histiocytic infiltration is visible.</td></tr>
<tr><td>10</td><td>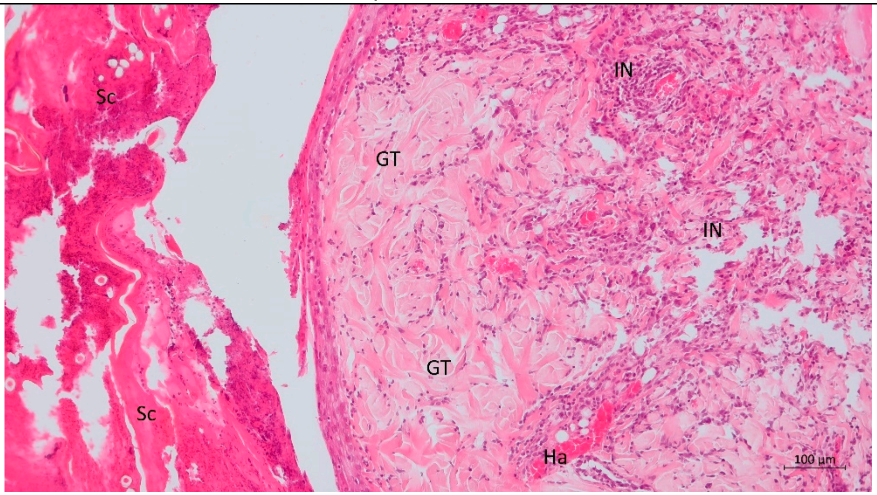
</td><td>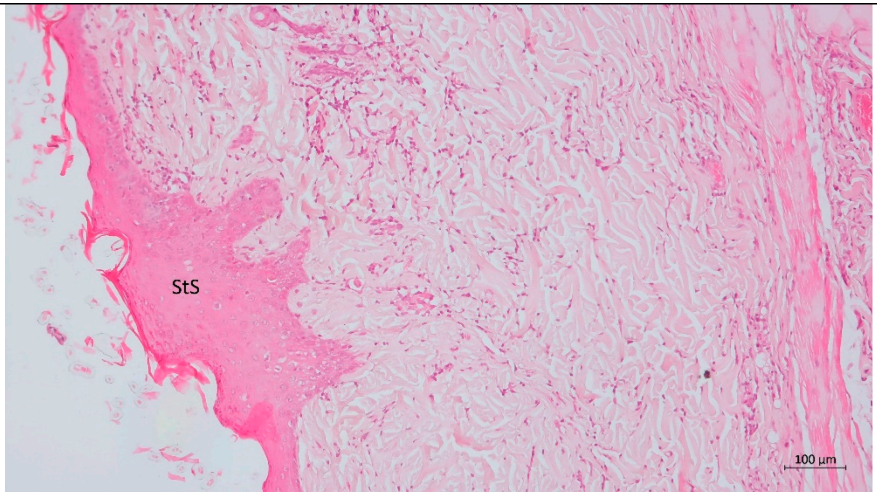
</td></tr>
</table>

| | | |
|---|---|---|
| | Weak epidermis, visible long epidermal protrusions, which, however, do not cover the entire wound surface. The entire surface of the wound is covered with a rich-cell serous clot. The clot is dominated by inflammatory cells - neutrophils. In the clot itself, cavities filled with eosinophilic, homogeneous exudate and hair residues are visible. Very rich granulation tissue is visible, especially on the edges of the wound. The wound center is dominated by a mixed infiltrate dominated by lymphocytes and histiocytes, which covers the dermis, subcutaneous tissue and muscles. Lack of any signs of healing, probably due to bacterial overweighing. | The wound is easy to identify. Advanced repair and healing processes. The entire wound surface is covered with young epidermis. In the epidermis, the expansion of the spinous layer is visible with the normal course of the keratinization process. A horizontal collagen bundle system dominates, with a slight activation of endomysial fibrocytes. No visible inflammatory infiltrate |
| 7 - Blank | 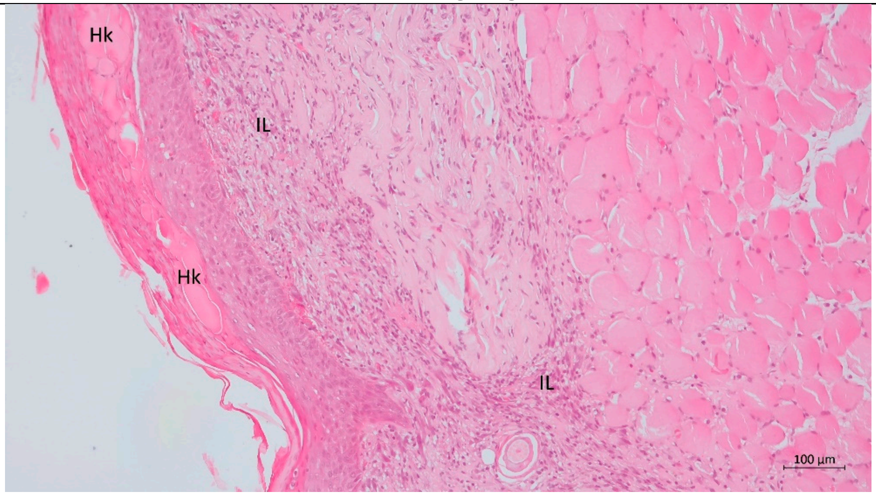 | 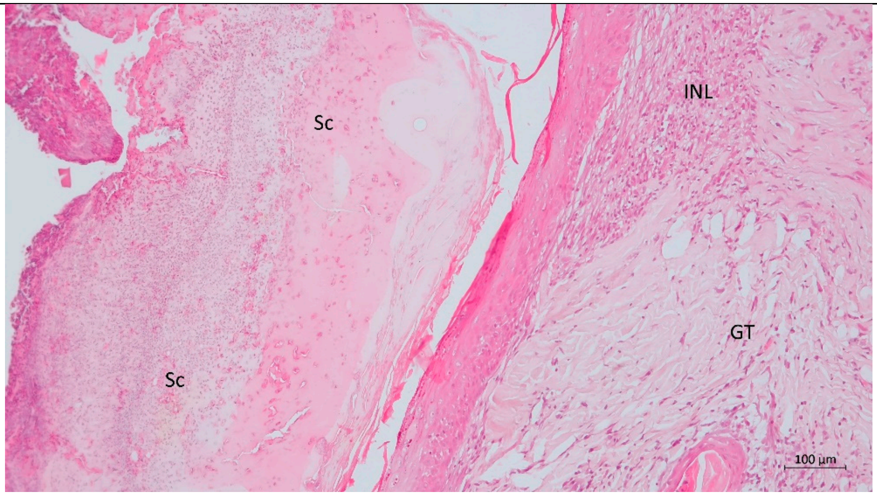 |
| | Visible growing epidermis on the wound niche, which does not fully cover it. The epidermis shows signs of rejuvenation with an extended granular layer. Slight hyperkeratosis with detachment of the stratum corneum. In the dermis, a moderate lymphocytic infiltration is visible - it is located both in the reticular and papillary layers - subepidermally, but it does not infiltrate the epidermis, it is focal. Collagen has a mixed structure and a mixed course. No hair follicles. | Wound niche visible. I am characterized by a thin layer of skin in which the epidermis predominates. In turn, the epidermis is dominated by a broadened spinous and focally granular layer. The stratum corneum is thin and in the superficial layers it becomes weaker. The dermis is made of loose, young collagen with a chaotic course with a visible moderate mixed neutrophilic and lymphocytic infiltrate. A focus of young connective tissue is visible with numerous fibroblasts and a mixed infiltrate - a focus of granulation tissue. The entire wound is covered with a scab adhering to the epidermis, composed of clotted serous secretion with rich purulent cellular detritus. This scab may have hindered the healing process. No visible vessels or emerging hair follicles. |

| | | | |
|---|---|---|---|
| Two weeks | 4 | *no data available* | 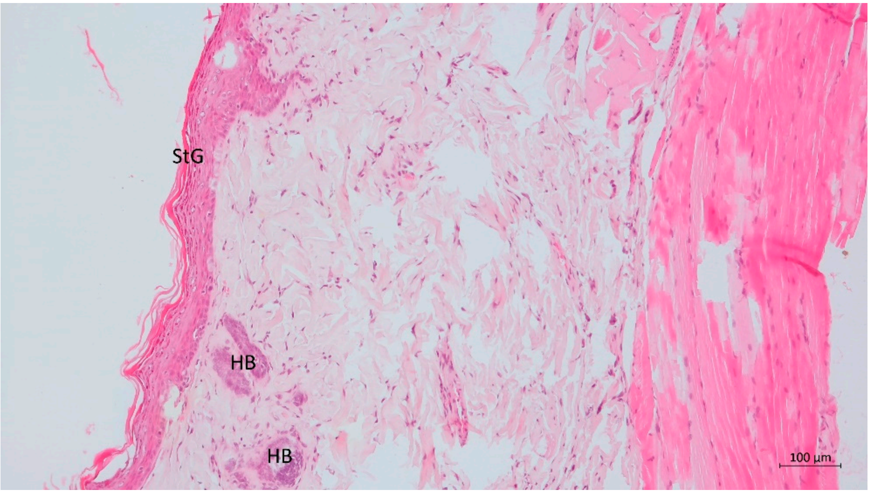<br> |
| | | The entire wound niche is covered with epidermis. In the epidermis, dilatation of the spinous (weak to moderate) and granular (moderate to significant) layers is visible. In the granular layer, numerous granules and strongly basophilic cytoplasmic drops are visible, even occupying entire cells. Visible clear acidophilic light layer - perhaps extended. Connective tissue of the dermis composed of reticular collagen and in the form of bundles with a mixed course. There is a mild to moderate inflammatory infiltrate, lymphocytic with a histiocytic component. No visible hair follicle buds. | The lower part of the induced wound is visible covered with young epidermis. The structure of the epidermis and the process of keratinization are normal (slight widening of the spinous layer and the outline of the granular layer. Collagen in the form of bundles with a mixed course. Weak lymphocyte infiltration in the deeper layers of the reticular layer of the dermis. On the edges of the wound, hair follicle buds are visible. |
| | 5 | 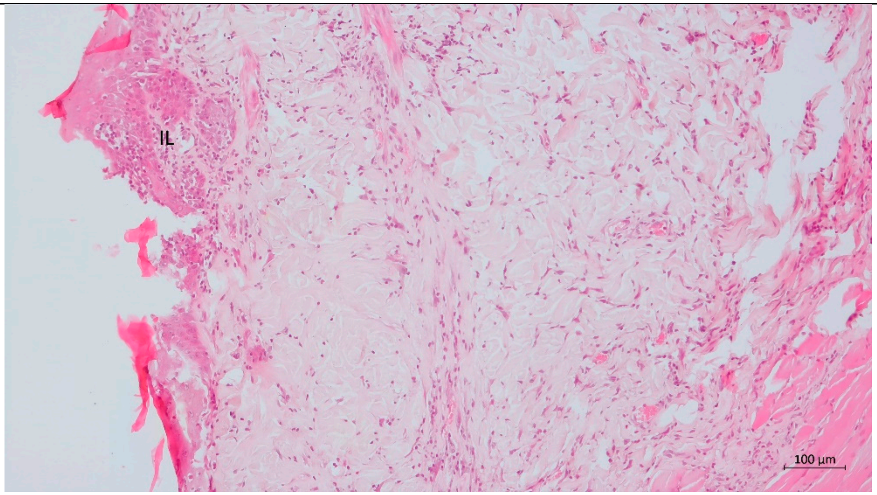<br> | 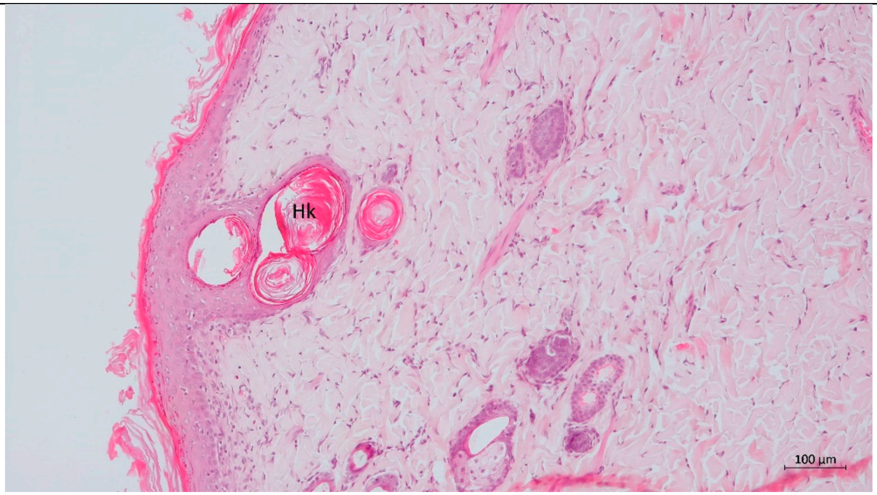<br> |

| | | | |
|---|---|---|---|
| | | A clear wound niche is visible, covered with fully young epidermis with a moderately widened spinous layer and normal keratosis. Clear light layer and practically invisible keratinization layer, which was probably mechanically torn off. Collagen fibers form bundles of mixed course. In the deeper layer of the reticular layer of the skin, there is a weak lymphocytic infiltrate and clear young capillaries. The changes described are characterized by a late healing process with ongoing connective tissue remodeling and descending, mild inflammation. | The wound niche is covered with young epidermis with a broadened spinous layer, a distinct granular layer with large and numerous granules, and an eosinophilic, extended clear layer. The keratinization process is normal and shows some features of hyperkeratinization. Visible delamination between the cells of the spinous layer, with moderate exudation. In the basal and spinous layers, there is a visible infiltration of mononuclear cells, predominantly lymphocytes. Collagen forms bundles of fibers that are predominantly horizontal. In the dermis, an inflammatory lymphocytic infiltrate is visible. Visible cell-rich focus of activated fibroblasts, lymphocytes with numerous capillaries - structure of granulation tissue. |
| | 6 | 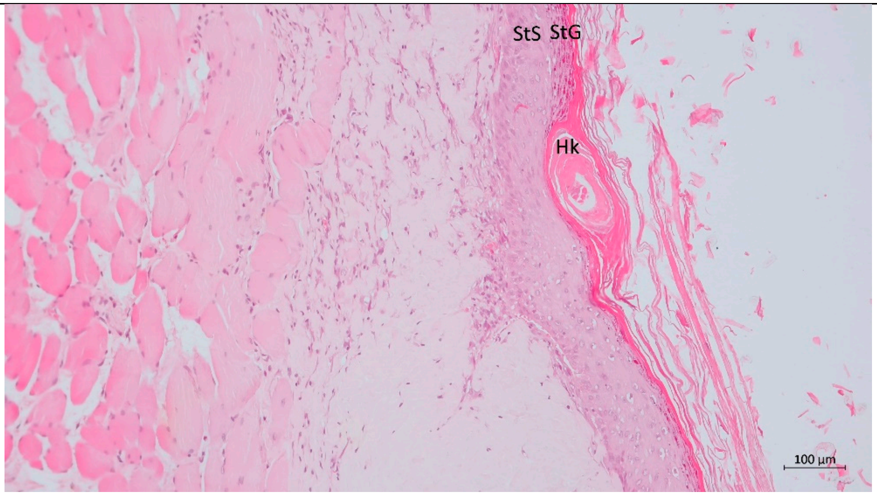  | 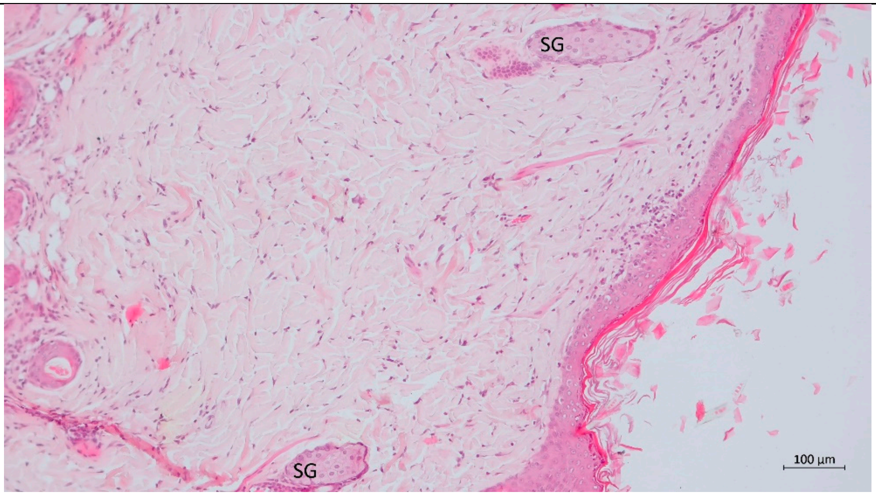  |
| | | The entire wound niche is covered with epidermis. The young epidermis is thickened - expansion of the spinous and granular layers. Visible hyperkeratinization with stratum corneum cysts with thick, homogenous, eosinophilic fluid. The layer peels off significantly. Visible young collagen with a mixed course, partly with a reticular structure. A faint lymphocytic-histiocytic infiltrate is visible in the dermis. Young capillaries are visible in the lower part of the reticular layer. No visible hair follicle buds. | Lower wound difficult to identify. It is distinguished from the surrounding skin by a slightly thicker and rejuvenated epidermis (with a slight fasting of the basal and spinous layers), the absence of hair, but with numerous young hair follicles visible. The arrangement of collagen fibers, architecture and vascularity of the dermis is almost identical to that of the surrounding skin. |

| | | | |
|---|---|---|---|
| | 11 | 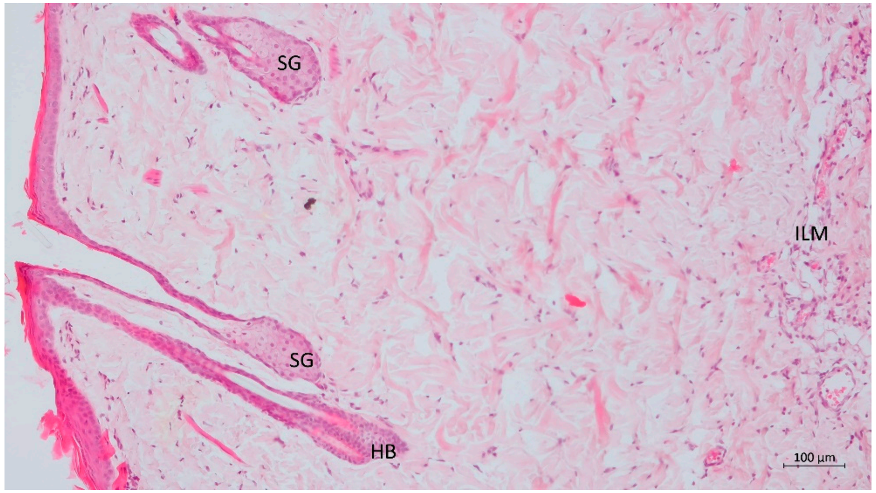  | 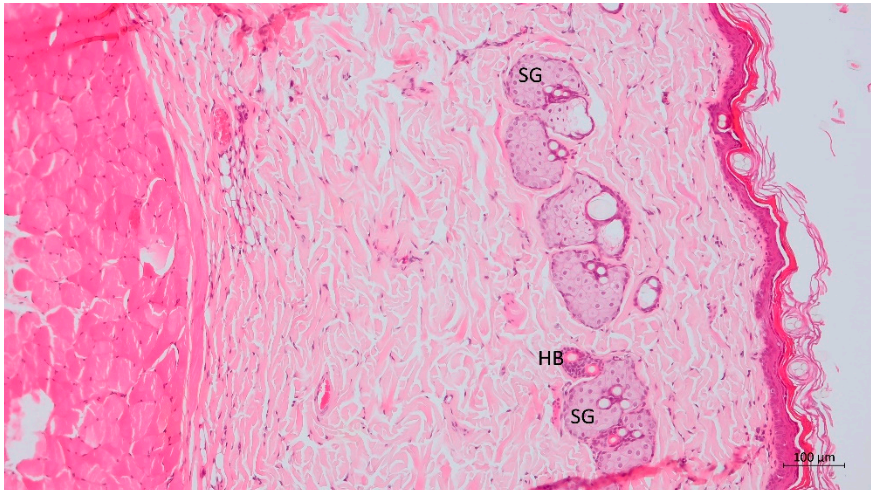  |
| | | A wound difficult to identify, with an advanced healing process. Visible normal epidermis and dermis. In the epidermis, the spinous and granular layers are slightly enlarged with normal keratosis. In the dermis, especially in the reticular layer, visible remodeling with a mixed arrangement of bunches of maturing collagen. The collagen system is mainly reticular. Visible hair follicles and sebaceous glands. Weak to moderate infiltration of mononuclear cells (lymphocytes and histiocytes) is seen in the lower part of the reticular layer and in the muscle layer. | Wound difficult to identify - advanced healing process. Visible focus of epidermal thickening with widening mainly of the basal and spinous layers with preserved normal keratosis. Single migrating lymphocytes are visible in the young epidermis. Under the described epidermis, a chaotic course of bunches of maturing collagen fibers is visible. Poor hair follicles and accessory glands with rejuvenated cells. No inflammatory infiltrate. |
| | 12 | 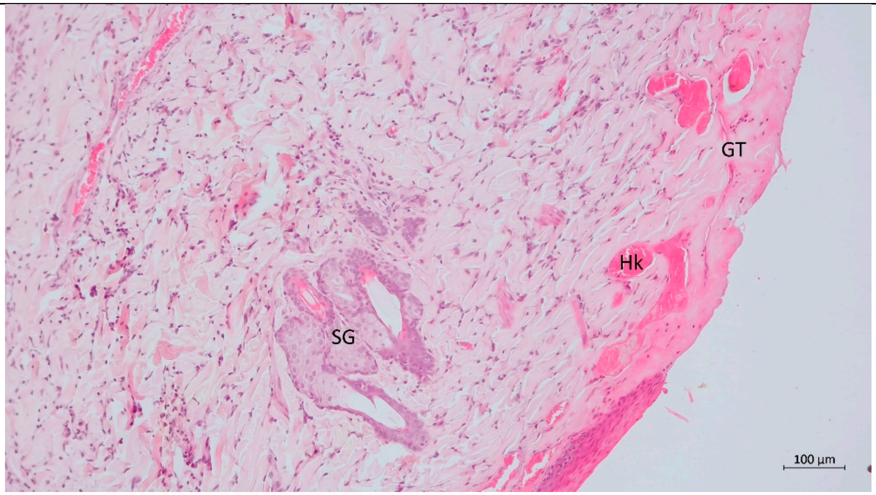  | 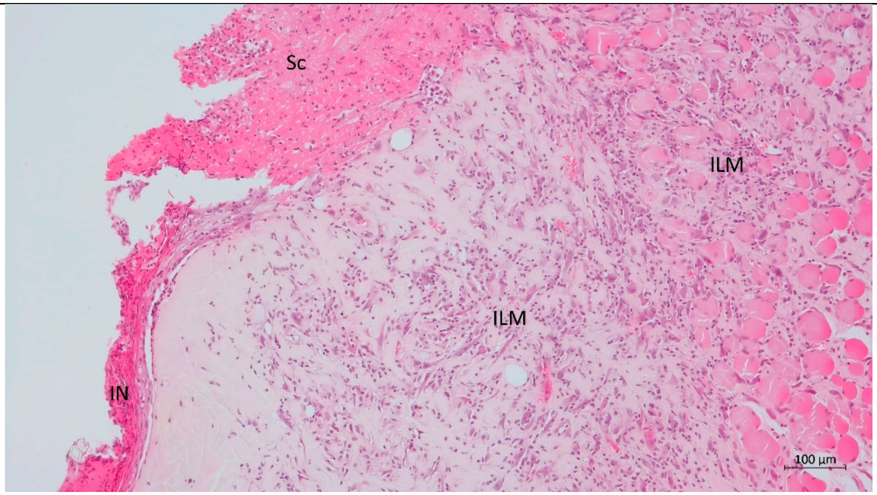  |

| | | | |
|---|---|---|---|
| | | The wound is easy to identify. Only partially covered by young epidermis - most of the wound is exposed by young collagen fibers. Young, growing epidermis with excessive hypertrophy of the stratum corneum, and in some cells visible nuclei. Thus, hyperkeratosis and parakeratosis can be identified as abnormalities of the young epidermis. The dermis is dominated by young collagen with a chaotic course. The dermis and muscle layer show a moderate, mixed inflammatory infiltrate with weak to moderate growth of granulation tissue. The blood vessels of the dermis are dilated and heavily filled with blood. | Most of the wound was covered with epidermis, which, however, was secondary destroyed by the serous clot on its surface and a strong purulent infiltration in the clot itself and in the papillary layer of the skin. The papillary layer is dominated by young collagen and exudative fibrin - with a mixed or vertical course. The reticular layer shows rich granulation tissue accompanied by a mixed infiltrate dominated by lymphocytes and histiocytes, also including the muscle layer. Visible features of earlier healing, which have been retarded, probably under the influence of bacterial overgrowth. |
| | 13 | 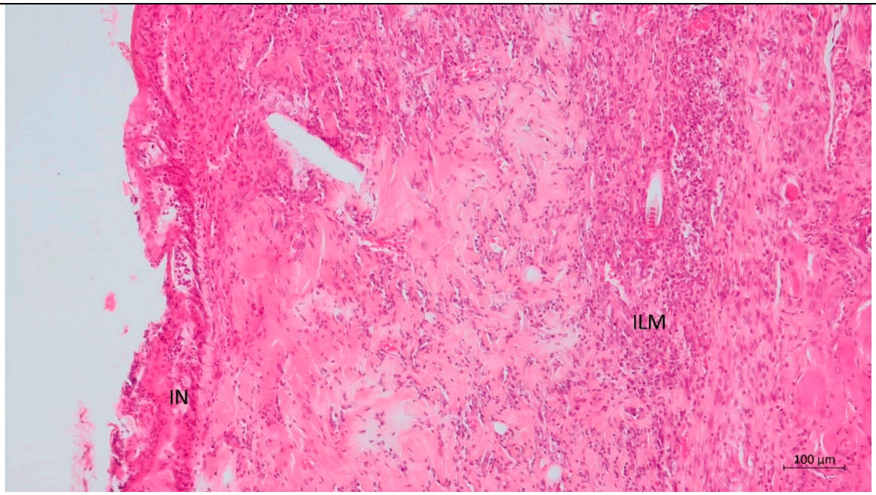 | 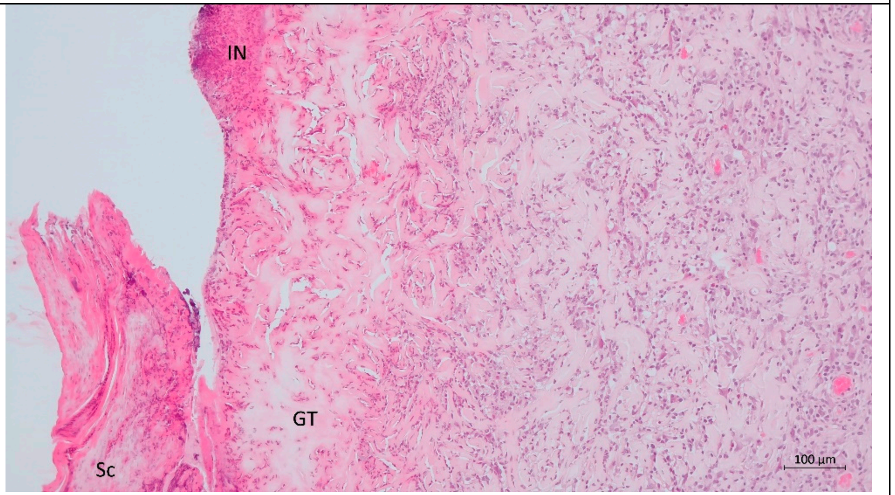 |
| | | The wound is easy to identify. Covered in large part with young epidermis. The epidermis is quite variable, from full thickness with an overgrowth of the spinous and basal layers to a single, two-celled layer of basal cells of the young growing epidermis. Cysts with exudative fluid and neutrophils and neutrophils infiltrating the epidermis are visible. A focus of clotted exudate with numerous neutrophils is also visible. No features in the epidermis of normal, complete keratosis. The dermis is dominated by young collagen with a mixed arrangement. A moderately abundant granulation tissue is visible, dominated by lymphocytic-histiocytic infiltration. Within this tissue there is a rich network of capillaries. | The wound is easy to identify. Covered in a small part with young epidermis - short epidermal protrusions. The surface of the papillary layer is composed of young collagen with a mixed course and a reticular structure. On its surface there are foci of purulent infiltration and detached/desquamated layers of moderate serous-purulent clot. The infiltration extends from the surface to the muscle layer and is purulent infiltration. Throughout the wound, the vessels are strongly dilated and filled with blood. The amount of granulation tissue is low to moderate. |

| | | | |
|---|---|---|---|
| | 14 - Balnk | *no data available* | 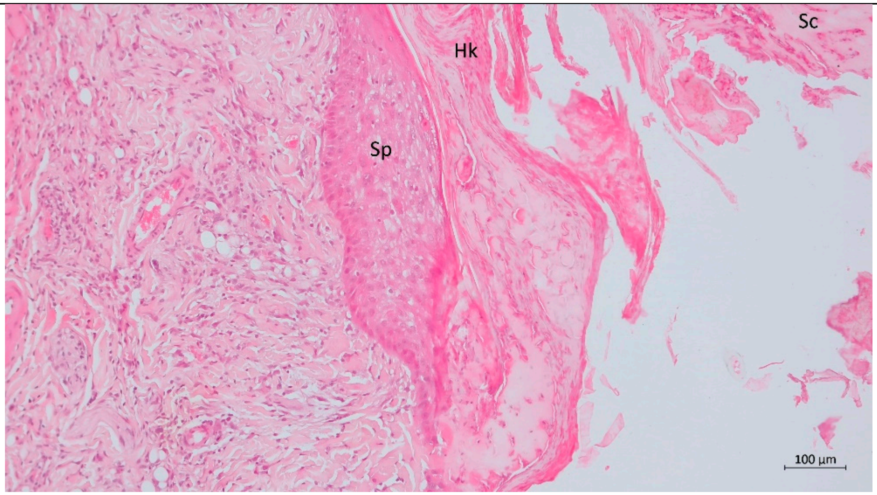  |
| | | Visible wound niche mostly covered with epidermis. In the epidermis, hypertrophy of the spinous layer and slight hyperkeratosis are visible. The epidermis is covered with a serous clot with a small amount of neutrophils. The dermis is dominated by young reticular collagen with a mixed course. Moderate infiltration in the dermis with lymphocyte predominance and weak to moderate granulation tissue with a very rich capillary network. | Visible wound niche, covered with young epidermis. In the epidermis, hyperplasia of the basal and spinous layers is visible. In the stratum corneum, there is a focus of hyperkeratosis (excessive keratosis), parakeratosis (presence of cell nuclei in the stratum corneum) and spongiosis (exudative fluid between keratocytes). The surface of the epidermis was covered with a fully exfoliated serous purulent clot. In the dermis visible bundles of maturing collagen with a mixed course. Weak stimulation of fibrocytes and weak lymphocytic infiltration in the reticular layer are visible. In the dermis, the buds of hair follicles and sebaceous glands are formed. |

**Explanation of markings in the images:**

StC – stratum corneum

StL – stratum lucidum

StG – stratum granulosum

StS – stratum spinosum

StB – stratum basale

HB – hair bulb

Hk – hyperkeratosis

Sp – spongiosis

I – inflammation

- L - lymphocytic
- M - monocytic
- N - neutrophilic

Oe – oedema

GT – granulation tissue

Ha – hyperaemia

SG – sebaceous gland

Sc – scab
